# Modelling the Geographical Range of a Species with Variable Life-History

**DOI:** 10.1371/journal.pone.0040313

**Published:** 2012-07-11

**Authors:** Sarina Macfadyen, Darren J. Kriticos

**Affiliations:** CSIRO Ecosystem Sciences, Canberra, Australia; University College London, United Kingdom

## Abstract

We show how a climatic niche model can be used to describe the potential geographic distribution of a pest species with variable life-history, and illustrate how to estimate biogeographic pest threats that vary across space. The models were used to explore factors that affect pest risk (irrigation and presences of host plant). A combination of current distribution records and published experimental data were used to construct separate models for the asexual and sexual lineages of *Rhopalosiphum padi* (Linnaeus) (Hemiptera: Aphididae). The two models were combined with knowledge of host plant presence to classify the global pest risk posed by *R. padi*. Whilst *R. padi* has a relatively limited area in which sexual lineages can persist year round, a much larger area is suitable for transient sexual and asexual lineages to exist. The greatest risk of establishment of persistent sexual and asexual populations is in areas with warm temperate climates. At the global scale the models show very little difference in risk patterns between natural rainfall and irrigation scenarios, but in Australia, the amount of land suitable for persistent asexual and transient sexual populations decreases (by 20%) if drought stress is no longer alleviated by irrigation. This approach proved useful for modelling the potential distribution of a species that has a variable life-history. We were able to use the model outputs to examine factors such as irrigation practices and host plant presence that altered the nature (transient or permanent) and extent of pest risk. The composite niche maps indicate pest risk in terms that are useful to both biosecurity agencies and pest managers.

## Introduction

Bioclimatic niche models and species distribution models that relate geographic observations of a taxa to environmental covariates have become an important modelling tool for addressing research questions in the fields of biogeography, conservation biology, invasion ecology, and evolution [1], [2], [3], [4]. The majority of bioclimatic models have sought to portray the current or potential distribution of species, rather than any other taxonomic level, though some effort has been applied to modelling species communities [5], [6]. When using bioclimatic niche models to estimate a taxon’s potential range, there is an implicit assumption that local adaptations in climate response within a species are encompassed within the modelled range. In most cases, the effect of modelling the species climatic range compared to modelling the range of a single genotype is likely to be a simple stretching of the climatic envelope to encompass the genotypes adapted to the climatic extremes. That is, the cold limits are set by the most cold-adapted genotypes, and the warm limits are set by the most warm-adapted genotypes. Where the species life-stages have significantly different climate responses, the bioclimatic models implicitly encompass the cold-, hot-, wet-, and dry-tolerant lifestages. Similarly, when modelling the niche of organisms such as some plant pathogens with complex life-histories involving multiple hosts, the model’s climatic parameters may simply span the requirements of two or more hosts (e.g. [7], [8]). Depending upon whether a particular life-history is facultative or obligate, the climatic requirements may be either additive or multiplicative, and different modelling approaches may be better or worse at capturing these responses.

Many pest aphid species exhibit complex life-histories involving multiple host plants [9]. The plasticity in their life-histories can involve different life-stages to cope with stressful conditions. Modelling such complex life-histories can be achieved through process-based population dynamics models or individual-based models applied to single locations (e.g. [10]). Unlike the challenge of encompassing a breadth of local adaptations, a single bioclimatic model that encompasses the geographical range of a species with multiple life histories may include significant areas that are climatically unsuitable for a particular life-history.

Most of the literature on species ranges has focussed on the conditions necessary for species persistence, although for some highly mobile, short-lived species, examples of ephemeral populations have been documented (e.g. [11], [12]). Such ephemeral populations often arise from species temporarily expanding their geographic range into regions that are only suitable for population growth during part of the year. Temporal variation in range boundaries has been acknowledged in the literature [13], and is an important factor influencing the occurrence of transient pest populations.

Here we focus on *Rhopalosiphum padi* (Linnaeus) (Hemiptera: Aphididae) (bird cherry–oat aphid), a pest of cereals in many countries around the world. Farmers rely heavily on the use of insecticides to control *R. padi* populations, particularly in winter wheat and barley crops across Europe, as it is a vector of crop diseases [14], [15], [16]. The widespread distribution and economic importance of *R. padi*, combined with a large number of published studies on the ecology and biology of this species, makes it an ideal subject for exploring how certain factors influence the risk posed by this pest. *R. padi* has a variable life-history. It alternates between host plants, with sexual individuals being produced once a year before moving onto the woody winter host (usually *Prunus padus*). In spring and summer individuals migrate to grass species (Poaceae) where they undergo parthenogenesis [17]. These asexual lineages switch to the sexual mode in response to a critical night length [18], [19] and produce frost-tolerant overwintering eggs [20], [21], [22]. In some regions, the sexual phase has been partially or completely lost, and obligate asexual lineages have developed [23]. These lineages do not alternate hosts and reproduce parthenogenetically on poaceous host plants throughout the year [17]. This life history leads to higher population growth rates during favourable winter conditions, but carries a risk of mortality during inclement winters [24]. *Rhopalosiphum padi* can form both apterous (wingless) and alate (winged) forms [25]. Its ability to produce alatae in response to photoperiodic and crowding stimuli facilitates access to seasonally abundant host resources [26], [27]. Viruses transmitted by *R. padi* can persist in grass host plants without causing symptoms and be transmitted to crops via feeding by aphids that have moved into emerging cereal crops [28], [29]. The viruses cannot persist in the woody winter host plant, so aphids emerging from overwintering eggs are not initially virus vectors [30]. Both sexual and asexual lineages can be present in a particular region during a season, but their relative abundance varies according to a variety of factors including winter climate, the phenology of host resources (e.g. the timing of cereal crop production), and the availability across space and time of the winter woody host plant [31], [32].

The currently documented range of *R. padi* includes areas of the world with persistent populations that are present year-round and regions with transient populations resulting from seasonal migration events. After considering the available documented life-history of *R. padi* we modelled the sexual and asexual lineages separately in order to capture information on persistent and seasonal transient populations. Here we use this novel modelling approach to describe the potential global distribution of each reproductive mode (sexual or asexual lineages) based on climatic conditions alone. We then recombine the results from these two models to understand the different types of pest risk posed by the organism throughout its potential range. We characterise the regional invasion risk by each reproductive mode, host presence, ability of the aphid to persist (permanent or transient), and the impact of land-use change (irrigated or dryland cropping). Through this process we illustrate how relatively complex life-histories can be incorporated into a simple niche model using an aphid species.

## Materials and Methods

### Current Distribution Records

**Figure 1:**
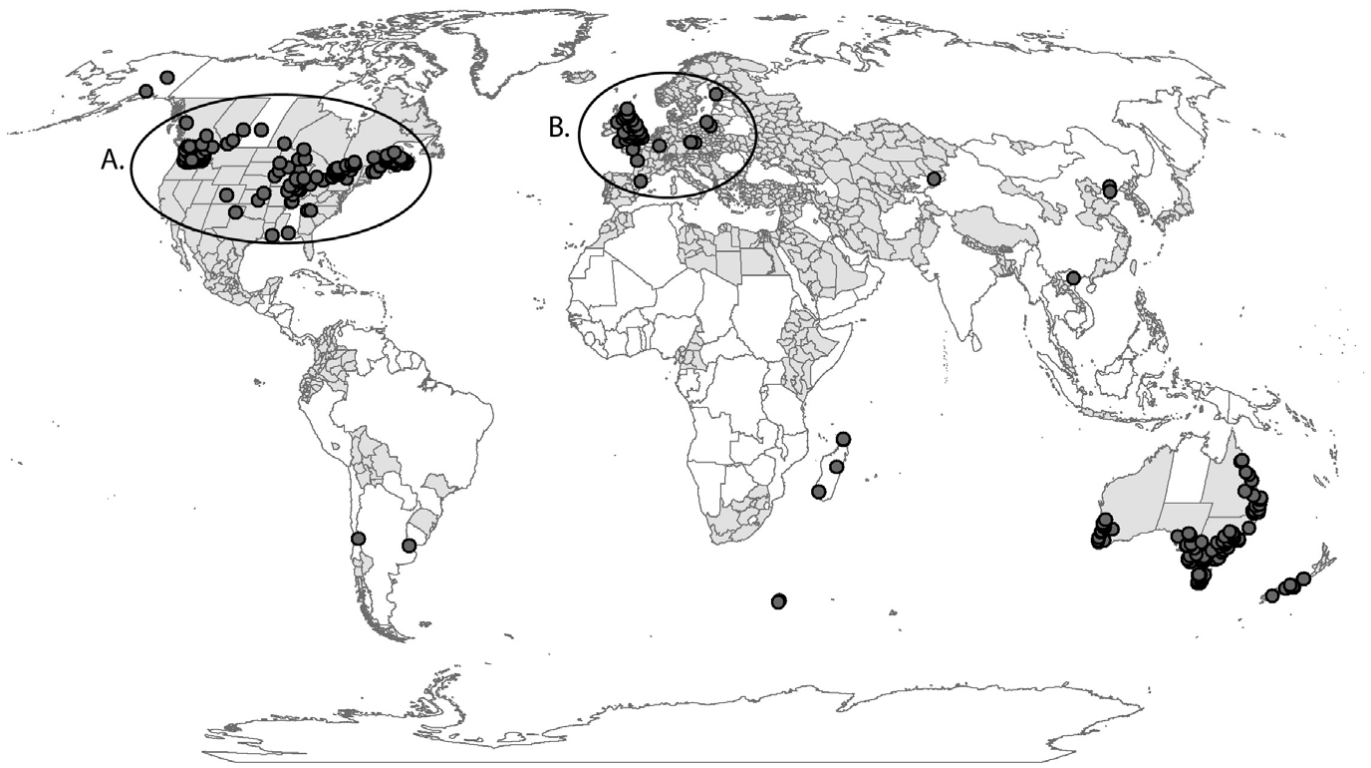
The world, showing point distribution records for the oat aphid *Rhopalosiphum padi*. The point records were collected from searches of the Global Biodiversity Information Facility, the Australian Plant Pest Database, published literature gathered through Web of Science searches, suction sample catches of the aphid throughout the UK and Europe (EXAMINE network), and specimen collection labels in museums (Australian National Insect Collection, Canadian National Collection of Insects). The CABI distribution map (Commonwealth Agricultural Bureaux International 1971) produced in 1971 and the Crop Protection Compendium (Commonwealth Agricultural Bureaux International 2007) was used to supplement these point locations with administrative region reports (shaded grey). The North American distribution points (circle A.) were used to fit both models, and the European distribution data (circle B.) used to verify the fit of the models.

A literature review was conducted on the biology and ecology of *R. padi* using a range of data sources focussing on life-history parameters that we considered important for *R. padi* presence and pest status around the world. The present known distribution of *R. padi* was estimated from a search of the Global Biodiversity Information Facility (www.gbif.org), the Australian Plant Pest Database (www.planthealthaustralia.com.au), published literature gathered through Web of Science searches (e.g. [15], [33], [34]), suction sample catches of the aphid throughout the UK and Europe (Harrington et al. 2004; EXAMINE network, www.rothamsted.ac.uk/examine/), and specimen collection labels in museums (Australian National Insect Collection, Canadian National Collection of Insects). These searches revealed point locations as indicated by latitude and longitude coordinates, and in some case only placenames (Fig. 1). In the latter case, for Australian sites, the Geoscience Australia Place Name search (www.ga.gov.au/map/names/) was used to link reported placenames with coordinates. For all other sites Google Earth® was used to gather coordinates. Prior to use, location records with erroneous geocodes or placenames that could not be reliably linked to coordinates were discarded. Subsequently, during model fitting, additional erroneously geocoded records were discovered where biologically unreasonable parameter values were required to fit outlying locations. In many cases, investigation of the descriptive locality information revealed that these records had the incorrect signs on their longitude or latitude value, and were altered accordingly. The point records generally give no indication as to the types of lineages (sexual, asexual or obligate asexual) represented by the collected specimen(s). Therefore, this point distribution map (Fig. 1, Fig. S1) was used as the starting point for model development, but experimental data from the scientific literature were used to distinguish between the sexual and asexual models. The CABI distribution map (Commonwealth Agricultural Bureaux International, 1971) produced in 1971, and the Crop Protection Compendium (Commonwealth Agricultural Bureaux International, 2007, *R. padi* entry updated 20 April 2001) were used to supplement these point locations with administrative region reports (Fig. 1, Fig. S1). These reports were used to code a world administrative region shapefile (ESRI, Redlands, CA) as present (1), or absent (0). These fuzzy spatial data were used to check model concordance, but were otherwise not used to fit model parameters.

*Prunus padus* is the main winter host plant in Europe, but *R. padi* can also use *P. spinosa* [35], *P. virginiana* and *P. pennsylvanica* in the USA [9], [23], and *P. cerasus* [36], although it is listed as non-preferred). There is some suggestion in the literature that *R. padi* can persist on *P. persica* in Asia (citation in [37]) however we could not confirm this report. The distributions of the five recorded *Prunus* host species were determined using GBIF point records and the USDA PLANTS database (USDA 2010, www.plants.usda.gov). These reports were used to code a world administrative region shapefile (ESRI, Redlands, CA) as present (1), or absent (0) if none of the five *Prunus* species were recorded as present in the region. For New Zealand extra data were gathered that showed that commercial cherries (including *P. cerasus*) are grown in the Otago, Marlborough and Hawkes Bay regions. These regions were coded as present (1) in the shapefile.

### Model-fitting Procedure

For this study, we chose to use CLIMEX [38], a climate-based computer system for exploring the relationship between climate, species distributions and patterns of growth. Whilst there is a plethora of techniques available for modelling species distributions [3], [39], CLIMEX allows us to incorporate information from various knowledge domains besides the geographic point locations at which the species has been found, and is useful for estimating the potential range of species in novel climates such as may be encountered in intercontinental model projections [40]. This latter ability is aided by the fact that it’s environmental response functions are constrained to conform with ecological principles such as the Law of Tolerance [41] and the Law of the Minimum [42]. CLIMEX was used to build models of each of the sexual and asexual lineages of *R. padi* (Table 1, Supporting Information S1). The asexual *R. padi* model was fitted first, relying upon a combination of geographic and published experimental data. The asexual *R. padi* CLIMEX model parameters were then adjusted to accommodate changes in the climatic tolerances documented for the sexual lineages. The asexual model makes no distinction between facultative asexual lineages and obligate asexual lineages that have lost their ability to reproduce sexually. The sexual model represents a life-cycle in which individuals will produce cold-resistant eggs to withstand cold winter conditions (obligate diapause). In areas where the winter is mild populations can still persist year-round but do not go into diapause. Both models were fitted to the North American distribution data (where we had comprehensive records), and verified using European distribution data (Fig. 1). Distribution data elsewhere were reserved for model validation. CLIMEX calculates a weekly Growth Index (GI_W_) that describes the species population response to temperature and soil moisture through the Temperature (TI) and Soil Moisture (MI) indices respectively (Table 1, Supporting Information S1). GI_W_ is integrated to provide the Annual Growth Index (GI_A_). Factors that limit a species’ ability to persist at a particular location are known as stress indices (hot, cold, wet, dry) and interaction stress indices (hot-wet, hot-dry, cold-wet and cold-dry). These individual stress values are combined to create the annual Stress Index (SI), and when combined with the Annual Growth Index (GI_A_) the programme calculates the Ecoclimatic index (EI). The EI is a measure of the overall suitability of a location for species persistence year-round (the larger the value the more suitable) [43]. The geographic range of a species is relatively insensitive to changes in parameters for growth indices; whereas the spatio-temporal pattern of relative climatic suitability within the geographic range is strongly affected by growth index parameters. In the absence of relative abundance measures across a range of sites it is necessary to draw upon observations of a species response to climatic factors in development rate experiments, or to infer parameters from phenological observations. Ideally, information from both of these sources can be used as a powerful form of independent cross validation.

**Table 1:** CLIMEX parameters for asexual and sexual reproductive forms of *Rhopalosiphum padi*.

Index	Parameter	Reproductive form		Units†
		Asexual	Sexual	
Temperature	DV0 = lower threshold	6	6	°C
	DV1 = lower optimum temperature	22	22	°C
	DV2 = upper optimum temperature	26	26	°C
	DV3 = upper threshold	30	30	°C
Moisture	SM0 = lower soil moisture threshold	0.1	0.1	
	SM1 = lower optimum soil moisture	0.5	0.5	
	SM2 = upper optimum soil moisture	1	1	
	SM3 = upper soil moisture threshold	1.4	1.4	
Diapause	DPD0 = threshold daylength to initiate diapause	**–**	**13**	Hours
	DPT0 = threshold minimum temperature to initiate diapause	**–**	**4**	°C
	DPT1 = threshold minimum temperature to end diapause	**–**	**4**	°C
	DPD = minimum number of days for diapause completion	**–**	**56**	Days
	DPSW = summer/winter switch	**–**	**0**	
Cold Stress	TTCS = temperature threshold	**−5**	**−10**	°C
	THCS = stress accumulation rate	**−0.001**	**−0.001**	Week^−1^
	DTCS = cold stress day-degree threshold (accumulates if theweekly number of degree days above DV0 is below this value)	**8**	**–**	°C days
	DHCS = cold stress degree-day accumulation rate	**−0.001**	**–**	Week^−1^
Heat Stress	TTHS = temperature threshold	32	32	°C
	THHS = stress accumulation rate	0.01	0.01	Week^−1^
Dry Stress	SMDS = threshold soil moisture	0.1	0.1	
	HDS = stress accumulation rate	−0.25	−0.25	Week^−1^
Wet Stress	SMWS = soil moisture threshold	1.4	1.4	
	HWS = stress accumulation rate	0.001	0.001	Week^−1^
Length of growing season	PDD = degree-day threshold (minimum annual total no. degree-days above 6°C (DV0) needed for population persistence	150	150	°C days

Differences in parameter values for the two forms are emboldened. Further information regarding the literature that was used to derive these parameters can be found in Supporting Information S1.

† Values without units are dimensionless indices.

N.B. Soil moisture indices are fractions of a 100 mm single-bucket soil moisture model where 1 is equivalent to field capacity, and 0 is oven dry. A value of 0.1 is approximately permanent wilting point.

Temporal patterns in abundance from suction samples ([44] EXAMINE trap network www.rothamsted.ac.uk/examine/) at three sites in Europe (Cáslav in the Czech Republic, Jokioinen in Finland and Rothamsted in England) were used to verify the fit of both models. The monthly catch of *R. padi* (males plus females) in suction samples at each site was averaged across the years 1996–2000. These data were compared to the average GI_W_ for each month produced by the CLIMEX model at each of these sites. This was calculated by totalling the GI_W_ values and dividing by the number of weeks in each month. In Australia, phenological data from monthly yellow pan collections from four sites (Canberra, Australian Capital Territory; Mackay, Queensland; Grove, Tasmania; and Adelaide Hills, South Australia) in 1961 were used in model comparisons [33]. Ideally we would like to compare phenological data collected across multiple years from these sites to capture inter-annual variability; however this information was not available. These data were compared to the average GI for each month produced by the CLIMEX model at each of these sites. Only the asexual model was assessed in Australia because sexual lineages are unlikely given that cold winter climates are limited, the primary wood host plant is rare (commercial cherries, *Prunus cerasus* are grown only in limited areas and there are a few point records for the other *Prunus* host species), and the timing of predominately winter cereal cropping throughout south-eastern and Western Australia means that asexuals would generally out-number sexuals.

### Classification of Pest Risk

The definitions of invasion risk adopted by the FAO in the International Standard for Phytosanitary Measures (ISPM) [45] provide a framework for the invasion risks assessed here. Areas are *endangered* if “…ecological factors favour the establishment of a pest whose presence in the area will result in economically important loss” (p 49), where *establishment* is defined as “The perpetuation, for the foreseeable future, of a pest within an area after entry” (p 49). In CLIMEX terms, we interpret those areas to be where the EI is greater than 0, indicating that all climatic factors necessary for survival have been met, albeit at a marginal level if the EI value is low (e.g. EI<10) and a suitable prunus host was present. A *transient* threat is posed if “the presence of a pest … is not expected to lead to establishment (p 56). This corresponds to an area where the EI is 0, and the GI_A_ is greater than 0. This rationale was used to create a matrix of invasion risk categories based on the spatial patterns in EI versus GI_A_ for each model, and the presence or absence of the primary *Prunus* host (Table 2). An area was considered potentially endangered if sexual populations could exist there year round, but a suitable *Prunus* host was reportedly absent. This extra risk category was introduced to account for uncertainty surrounding the *Prunus* distribution records (in some areas the *Prunus* host may be present but has not yet been recorded in the databases we accessed). The risk classifications were applied in Excel using a set of nested ‘IF THEN’ conditions.

**Table 2:** Classification of invasion risk categories based on the International Standard for Phytosanitary Measures (ISPM) (FAO 2006) used for the classification of *Rhopalosiphum padi*.

Code	Description	EI		GI		Generations†	*Prunus* Host#
		Asexual	Sexual	Asexual	Sexual		
**1**	Endangered by Asexualonly and transient sexual	>0	0	–	–	–	–
		>0					0
**2**	Endangered by Sexual onlyand transient Asexual	0	>0	–	–	–	1
**3**	Endangered by both	>0	>0	–	–	–	1
**4**	Transient by Asexual only	0	0	>0	0	>1	–
**6**	Transient by both	0	0	>0	>0	>1	–
**7**	Potentially Endangeredby Sexual only	0	>0	–	–	–	0

The ecoclimatic index (EI) and growth Index (GI) were calculated using two CLIMEX models that illustrate the asexual and sexual modes of reproduction.

† Number of generations - must have more than one generation per year.

# 1 indicates *Prunus* host is present, 0 indicates it is absent.

The ability of *R. padi* to invade an area seasonally where crops are grown under natural rainfall conditions was assessed by spatially intersecting the Australian distribution data for the asexual *R. padi* model, the cropping area polygons selected from the Land Use of Australia, V4, 2005–2006 dataset (Australian Bureau of Agricultural and Resource Economics and Sciences, www.abares.gov.au), and the CLIMEX results. Transient areas where seasonal invasion could take place were coded 1 and represented cells with GI_A_>0 and EI = 0. Endangered areas where asexual lineages could persist year round were coded 2 and represent cells with EI>0. In both cases, more than one generation per year was required.

### Climate Data

Baseline climatic data used to build the models was taken from the TYN CL1.0 dataset supplied by the Climatic Research Unit (CRU) of the University of East Anglia for the period 1961–1990 [46]. For the ‘Compare Locations’ analyses, CLIMEX requires five climatic variables in the form of long-term monthly means of daily minimum temperature, daily maximum temperature, relative humidity at 0900 hours and 1500 hours, and monthly total precipitation. Where necessary, the variables supplied by the CRU were transformed as described by Stephens *et al.* [47] and Kriticos *et al.* [48]. The output from the models (EI values, GI values and Risk categories), illustrated on global maps, were plotted at the 0.5° grid cell scale.

### Irrigation Scenario

The potential distribution of *R. padi* appears to be influenced by irrigation in Mediterranean climates, providing a means for it to persist through the dry summers [49]. To assess the potential influence of this agricultural practice we ran an irrigation scenario in CLIMEX and applied it to both sexual and asexual models. The irrigation scenario consisted of a simple “top-up” irrigation of 2 mm day^−1^ during the six summer months. In this case, for any week in which there is less than 14 mm rainfall, the deficit was added prior to the calculation of the soil moisture balance.

## Results

### Current Known and Modelled Geographic Distribution

**Figure 2:**
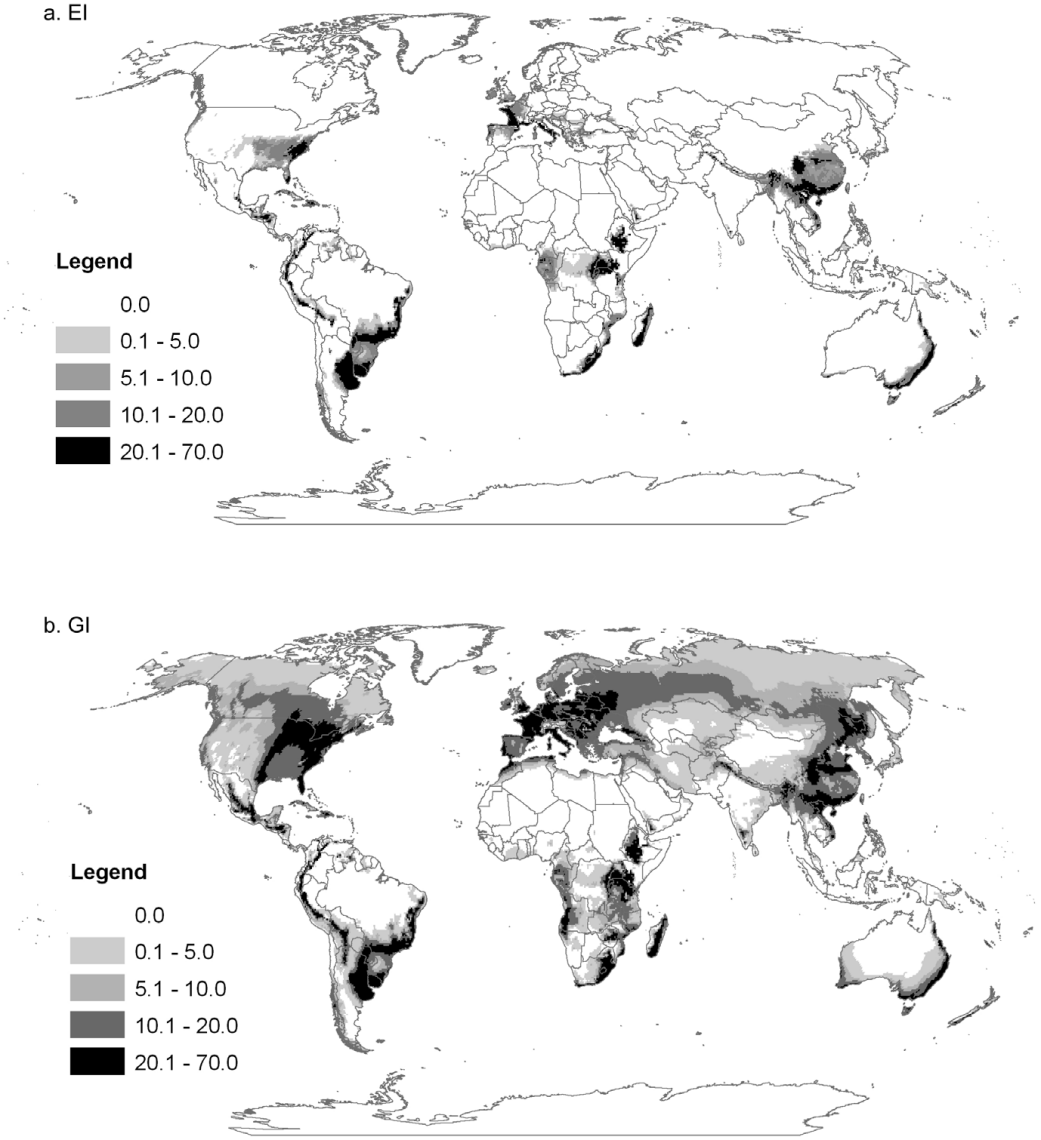
The world, showing the climate suitability for the oat aphid *Rhopalosiphum padi* estimated using a CLIMEX model. The model was parameterised for the asexual mode of reproduction and shows; (a), the Ecoclimatic Index, indicating the potential distribution of sexual populations (potential for year round persistence EI>0); and (b), the annual Growth Index GI_A_ indicating areas that are suitable for growth at some point during the year (GI_A_>0). This model does not include data about the presence of a suitable primary host plant.

**Figure 3:**
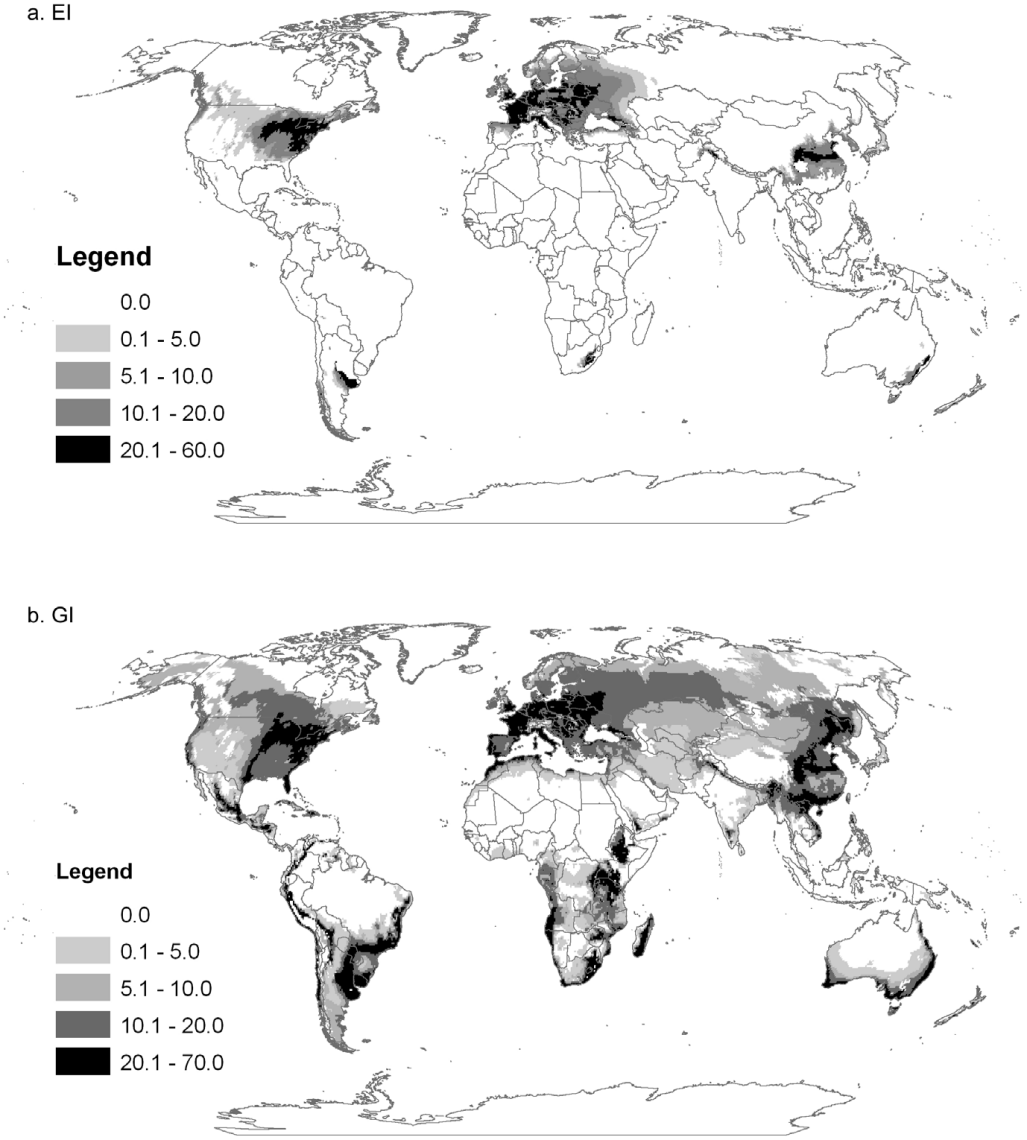
The world, showing the climate suitability for the oat aphid *Rhopalosiphum padi* estimated using a CLIMEX model. The model was parameterised for the sexual mode of reproduction and shows; (a), the Ecoclimatic Index, indicating the potential distribution of sexual populations (potential for year round persistence EI>0); and (b), the annual Growth Index GI_A_ indicating areas that are suitable for growth at some point during the year (GI_A_>0). This model does not include data about the presence of a suitable primary host plant.

*Rhopalosiphum padi* is a palaearctic species that appears climatically suited to large areas of both northern and southern hemispheres (Fig. 1). Both the sexual and asexual models (Fig 2 and 3 respectively) match the known distribution in its native range in North America, with little apparent model commission error (areas modelled as suitable without occurrence records). Elsewhere the models also fitted all known observations. The GI_A_ output of the asexual and sexual models matched well with the CABI distribution map produced in 1971 (compare Fig. 1 with Fig. 2b and Fig. 3b). Our model also highlights additional areas which appear to be climatically suitable for *R. padi* but were not recorded as having the species on the CABI map. However, the nature of the CABI distribution map means that these areas should not be interpreted as indicating model commission error, but rather that the model is untested in those areas. Further support for our models comes from the results of the temporal comparison of the yearly phenology graphs produced by the models and the empirical data from the EXAMINE traps and Hughes 1961 [33] yellow pan traps. Similar patterns in terms of months of peak abundance were found between the data sets (graphs not shown). The asexual model (Fig. 2) clearly illustrates the inability of *R. padi* to persist year round in areas with severely cold winters, however the GI_A_ suggests that many cropping areas around the world may nonetheless be suitable suitable for seasonal invasion (i.e. long-distance and local movement of asexuals from grass species into the growing cereal crop). The model suggests that sexually reproducing aphids (Fig. 3) can persist in areas with more severe cold winters than asexual populations (e.g. parts of Eastern Europe, Fig. 3a), however the geographic pattern of growth potential described by the GI_A_ is more restricted than in the asexual model due to the presence of an obligate diapause, during which growth is suspended. The sexual model does not contain information about the presence of primary woody host plant in the regions with cold winters, which is one factor crucial for successful development in sexual lineages. When we compared the global distribution of *Prunus* host plant point records we found that they encompassed the predicted distribution of *R. padi* based on our sexual model (EI>0). We would expect this to be the case given that we fitted the sexual model to the known distribution of *R. padi*, and adjusted the cold stress and diapause parameters according to evidence that sexual populations rarely occur in some regions of Canada.

### Pest Risk Around the World

**Figure 4:**
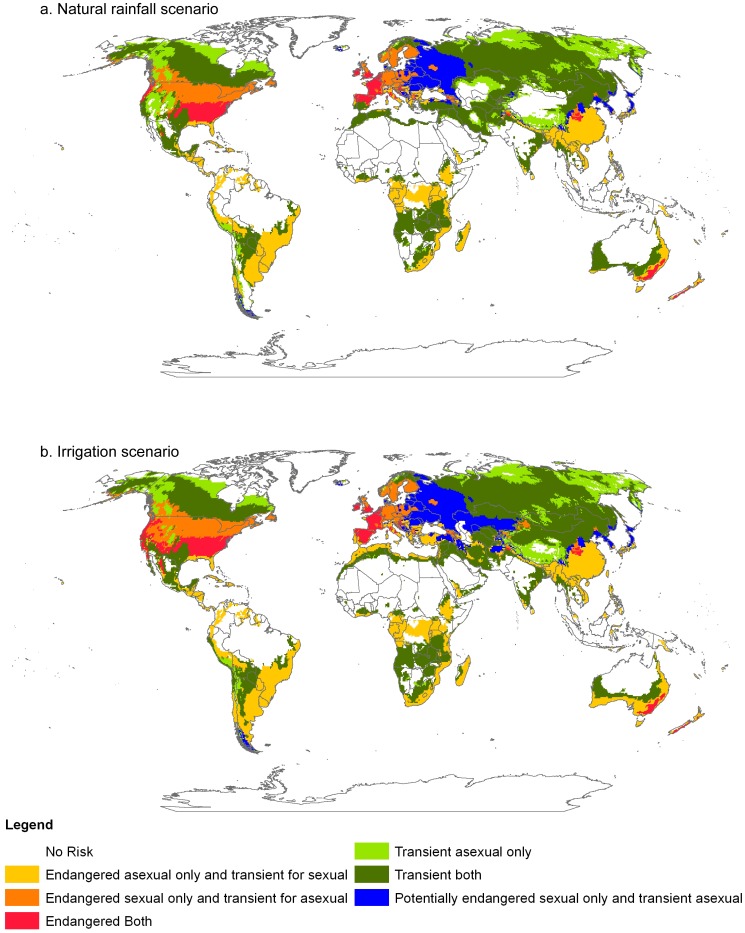
The world, showing risk categories for *Rhopalosiphum padi* invasion. Two scenarios are shown that represent; (a), natural rainfall conditions; and (b), an irrigation scenario. Results are based on the output from sexual and asexual CLIMEX models and the presence or absence of the primary woody host plant. Invasion risk categories are based on the International Standard for Phytosanitary Measures (ISPM) (FAO 2006). ‘Endangered’ indicates areas that are at risk of *R. padi* populations establishing and persisting year-round, ‘Transient’ indicates areas that are at risk of seasonal reinvasion but conditions are not suitable for persistence year-round, and ‘Potentially endangered’ indicates areas at risk of persistent populations year-round if a suitable *Prunus* host plant were introduced.

**Figure 5:**
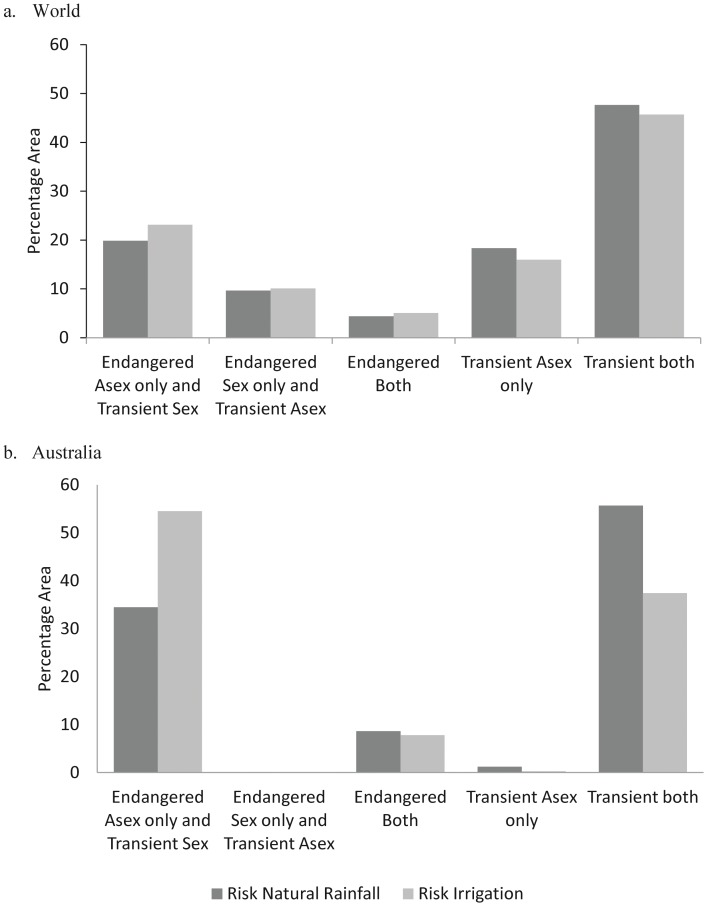
Proportion of area occupied by each *Rhopalosiphum padi* risk category. The data for the world (a), and Australia only (b), is generated from two CLIMEX models. The models illustrate the asexual and sexual modes of reproduction of the species, the presence or absence of the primary host plant and scenarios incorporating natural rainfall or irrigation. The ‘potentially endangered sexual only and transient asexual’ risk category is not shown here.

Data on the presence (or absence) of the *Prunus* host species were incorporated into the risk analysis to give a more comprehensive global picture of *R. padi* risk (Fig. 4). At the global level almost half of the area suitable for *R. padi* falls in the ‘transient both’ risk category (48% natural rainfall, 46% irrigation) (Fig. 5). The next most common risk category is for ‘endangered asexual only and transient sexual’ (20% natural rainfall, 23% irrigation). The regions of the world experiencing risk from establishment of populations of both the sexual and asexual lineages (‘Endangered both’) closely reflect warm temperate climates in North America, Europe and Australia. Areas that are transient for both sexual and asexual include continental climates with a cool summer (northern hemisphere) or arid climates (southern hemisphere) (Fig 4). Large areas of Eastern Europe (Russia) are potentially endangered by populations of sexual *R. padi* lineages (and transient asexuals) if a suitable *Prunus* host plant species is present in this area (or is introduced into this area). When an irrigation scenario was included, the greatest change in risk categories is seen in regions of the world with arid climates (Fig. 4). At the global scale, there is very little difference between natural rainfall and irrigation scenarios in terms of the proportional area occupied by each risk category (Fig. 5). However, when we examine more closely a country such as Australia (which is dominated by an arid climate, and only asexual lineages are common) there are quite dramatic differences between natural rainfall and irrigation scenarios (Fig. 5). In Australia, the amount of land endangered by populations of asexual lineages and at risk from transient populations of sexual lineages increased (by 20%) under irrigation and land suitable for transient lineages of both reproductive modes decreased under irrigation (by 18%).

**Figure 6:**
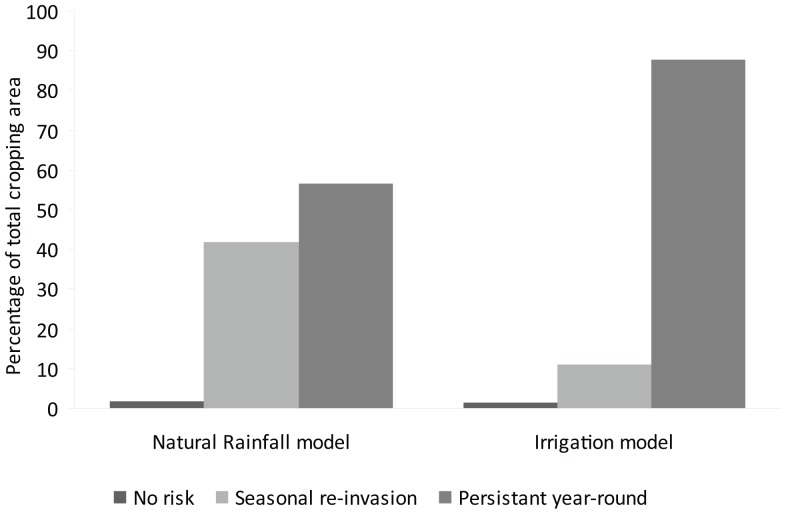
Proportion of total cropping area in Australia that is at risk of persistent year round populations of *Rhopalosiphum padi* or seasonally transient populations. Data generated by a CLIMEX model illustrating the asexual mode of reproduction in this species under natural rainfall conditions and incorporating an irrigation scenario.

For Australia we extracted the cropping regions from the Land Use V4 dataset to examine what proportional area of the cropping zone (approximately 14.6 million ha of land) was at risk from seasonal re-invasion by transient populations of asexual lineages. Whilst land outside the cropping zone is also climatically suitable for *R. padi* the impact of attack by *R. padi* on crops is of economic importance. Only the asexual model output was considered in this analysis. A very small amount of cropping land (1.6% or 232,000 ha) was unsuitable for growth of *R. padi* asexual lineages. When an irrigation scenario was applied, the area of land at potential risk from persistent year-round populations of asexual lineages of *R. padi* increased to 33% (Fig. 6). This equates to an extra 4.5 million ha of potential cropping land falling into this risk category.

## Discussion

Here we have used a relatively simple modelling methodology to describe the global potential distribution (based on climatic variables) and pest risk posed by an aphid species with a variable life-history. Using this approach we have been able to integrate information on differences in environmental limits to growth for the sexual and asexual lineages, along with the presence or absence of the primary host plant, to present global pest risk patterns. The resulting models accord with the current known geographic distribution of this species and provide comprehensive maps of the potential distribution in areas where collection records are scarce or unavailable. The areas modelled as being environmentally suitable for *R. padi*, but have no collection records provide the basis for a set of testable hypotheses concerning sampling biases, non-climatic factors (e.g., host distribution), wind patterns, invasion lags and model formulation. The model output provided insight into the nature and extent of the pest risk posed by this species, and how factors such as primary host presence and irrigation practices might significantly influence the extent and nature of the pest risk.

Asexuality generally limits the adaptive potential of a species when colonizing new areas (but increases colonisation potential). However, *R. padi*, like other pest aphids [50], occupies a broad climatic niche that has been crucial to its widespread invasion success around the world; indeed, most of the major grain growing regions of the world appear climatically suitable for this species. From our results we can see how the presence of the primary host plant and irrigation can significantly increase the potential range of this species. Using this methodology we can also see that large areas of the world are suitable for populations of *R. padi* to persist year-round. Whether or not a particular region is more suitable for sexual versus asexual lineages in our models, not surprisingly depends largely on cold winter conditions and the presence of an alternative primary host. The absence of this primary host plant limits the sexual lineages ability to occupy areas that have suitable conditions for growth at other times of the year. Clearly, if this primary host plant was more widespread we would see larger areas of land suitable for endangered sexual lineages (blue areas in Fig. 4). Our analysis does not include factors such as competition between sexual and asexual lineages throughout time, and colonisation history, which may influence the relative abundance of sexual versus asexual lineages in particular regions. Furthermore, there is some evidence that not all the *Prunus* host species we have included here are equally suitable as hosts [35].

The irrigation scenario results are particularly relevant to arid cropping areas such as those found in Australia. The model suggests that the majority of the wheat/sheep belt of Australia is favourable for the growth and survival of *R. padi*. In fact less than 2% of cropping land was modelled as unfavourable. Very few parts of the Australian wheat/sheep cropping belt are currently under irrigation (approximately 7% of total cropping area is irrigated). However, irrigation also occurs on land used for pastures and horticulture. The addition of an irrigation scenario to the asexual model made a larger area of cropping land suitable for persistent populations year-round.

The modelling results provide useful guidance for the future management of this pest. Firstly, the abundance of sexual and asexual lineages in a particular area has implications for plant damage due to the transmission of aphid-vectored viruses. Vitou & Edwards [51] showed that aphids (*Diuraphis noxia*) from an asexual lineage caused significantly more damage to wheat in comparison to a sexual lineage in a laboratory-based experiment. Cropping areas of the world that are currently suitable for the establishment of sexual lineages may experience a greater threat from *R. padi* vectored viruses if warmer winters in the future lead to a greater prevalence of asexual lineages [31], [52], and grassy weeds that act as alternative hosts serve as virus reservoirs. Furthermore, the differences in time of arrival and build-up of migratory asexual populations and local sexual populations may alter their relative impacts. Secondly, these types of niche models represent the first step towards predicting seasonal peaks in aphid abundance [53]. The climatic favourability of a particular site for growth of aphid populations as described through the GI_w_ indicates where outbreaks could potentially occur, but the timely arrival of migrants from suitable oversummering or overwintering sites is also necessary for the build-up of populations. More broadly, modelling the climate suitability for each *R. padi* reproductive mode separately enabled the geographic patterns of the different types of risk to be mapped, which can aid in the development of region-specific pest management plans.

As with all studies that aim to model a species with a complex life-history we have simplified parts of *R. padi*’s life-cycle which puts certain limitations on the results we discuss here. We have presented the asexual and sexual reproductive modes as two separate models, but in reality they are a continuation of the one life-cycle. Asexual clones can produce sexual clones in response to seasonal conditions (with the exception of obligate asexual lineages that generally will not alter their reproductive mode despite conditions). By then combining these two models to assess pest risk, our distribution maps reflect the complexity inherent in this species life-history across space but not across time. Whist it is possible to develop more complex lifecycle models (using tools such as DYMEX and ILCYM (research.cip.cgiar.org)) to generate pest risk models, their information demands are great, and it is likely that ecologically significant factors might remain unaddressed when the detailed model is applied across a gridded climate station network. That is, in an effort to achieve more precision, they may run into ecological scaling issues that make them less accurate. For this reason, a complementary modelling approach can prove very useful for the study of a species. A niche model can provide the broad geographical and phenological context [54] and a process-based population dynamics model [55] can provide the detailed insight into the system dynamics. In these examples with modelling the Queensland Fruit Fly, the CLIMEX model indicated overall climate suitability patterns, and was used for an assessment of invasion risk and climate change sensitivity [54]. The detailed population dynamics model was used to provide case study insight at selected locations where detailed information was available [55]. The starting point for both CLIMEX models was *R. padi* point distribution records collated from a range of sources (Fig. 1). Some time was spent removing (or corroborating) erroneous records, though we cannot rule out the possibility of incorrect identifications. Likewise there are large areas of the world that appear suitable for *R. padi* for which no point references were found. For example, caution must be exercised when interpreting the model output in tropical areas as *R. padi* may experience some form of biotic stress associated with hot and wet conditions. However, we did not have sufficient reliable data with which to fit parameters to explore this point. There is some evidence that aphid populations can be limited by the presence of parasitoids or predators in certain circumstances [56]. These biotic interactions are likely to affect the abundance of the species, but perhaps not restrict its potential range significantly. In regions such as South America and China, that appear climatically suitable, we found very few published records. It may be that there are many records for *R. padi* that have not yet been digitized (and so are not available) or a lack of recording effort in these regions. In contrast other regions (e.g. south-west Western Australia) have a disproportionate number of records that may represent a concerted research effort in this area. The CLIMEX programme allowed us to incorporate information from published experimental studies that examined growth and development parameters for *R. padi* however for parameters such as moisture index no published information was available (Supporting Information S1). In this case we took a conservative approach and used the wilting point of plants as a surrogate for moisture index. If *R. padi*, responds differently to moisture conditions than its hosts, this may alter the output of our models. Finally, the climatic variables obtained from weather stations used in this model may not reflect the microclimate experienced by aphids whilst on their host plant [57]. However, given the large spatial scale used in this study it is unlikely that microclimate issues will have a significant impact on the potential range of *R. padi* as mapped here. Despite these limitations our models describe some of the more important life-history parameters of *R. padi* and therefore are useful for investigating applied issues (seasonal movement, irrigation) and the broader pest management issues discussed above. The models showed that whilst *R. padi* has a relatively limited area in which sexual lineages can persist year round, a much larger area of land globally is suitable for transient asexual and sexual lineages to exist during favourable seasons. The modelling approach proved useful for examining factors that influence the seasonal growth and potential range of a pest species that occupies a broad climatic niche and has a variable life-history. Whilst niche models cannot incorporate all the complexity inherently present in any single species, understanding species ecology and thereby including the right complexity into the model goes some way to making these models useful in applied contexts.

## Supporting Information

Figure S1 **Global recorded distribution of** ***Prunus*** **host plants of** ***R. padi*** **.** The distributions of the five recorded *Prunus* host species were determined using GBIF point records and the USDA PLANTS database (USDA 2010, www.plants.usda.gov) (a). These reports were used to code a world administrative region shapefile (ESRI, Redlands, CA) as present (1), or absent (0) if none of the five *Prunus* species were not known to occur in the region (b). For New Zealand extra data were gathered that showed that commercial cherries (including *P. cerasus*) are grown in the Otago, Marlborough and Hawkes Bay regions.
(DOCX)

Supporting Information S1 **More information on the parameterization of CLIMEX models.**
(DOCX)
