# Supplementary figures and images for: Modelling the Geographical Range of a Species with Variable Life-History
Source: PLoS One. 2012 Jul 11;7(7):e40313. doi: 10.1371/journal.pone.0040313 (PMC3394791; doi:10.1371/journal.pone.0040313)

**Supporting Information Figure S1**

**a.**
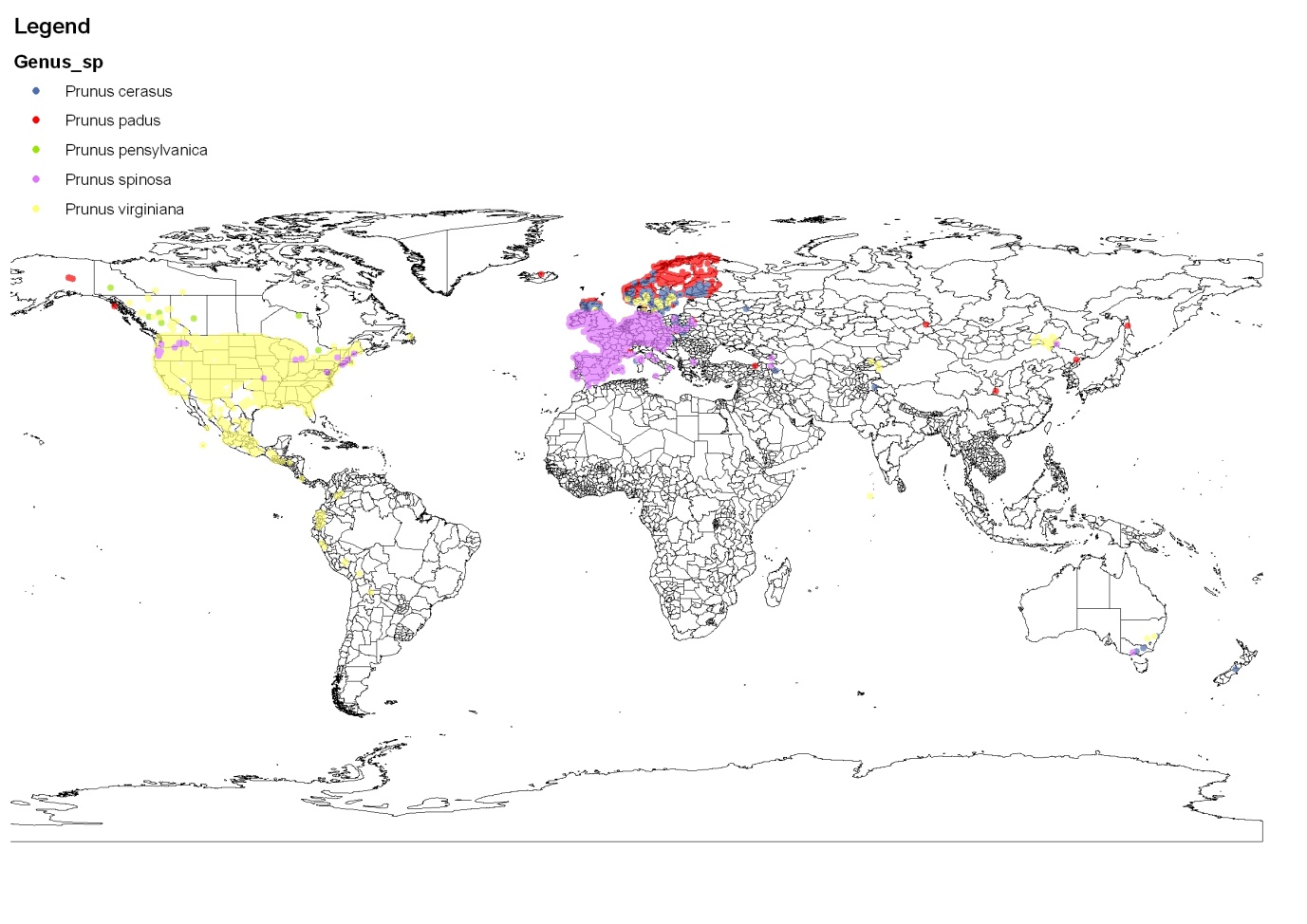
**b.**
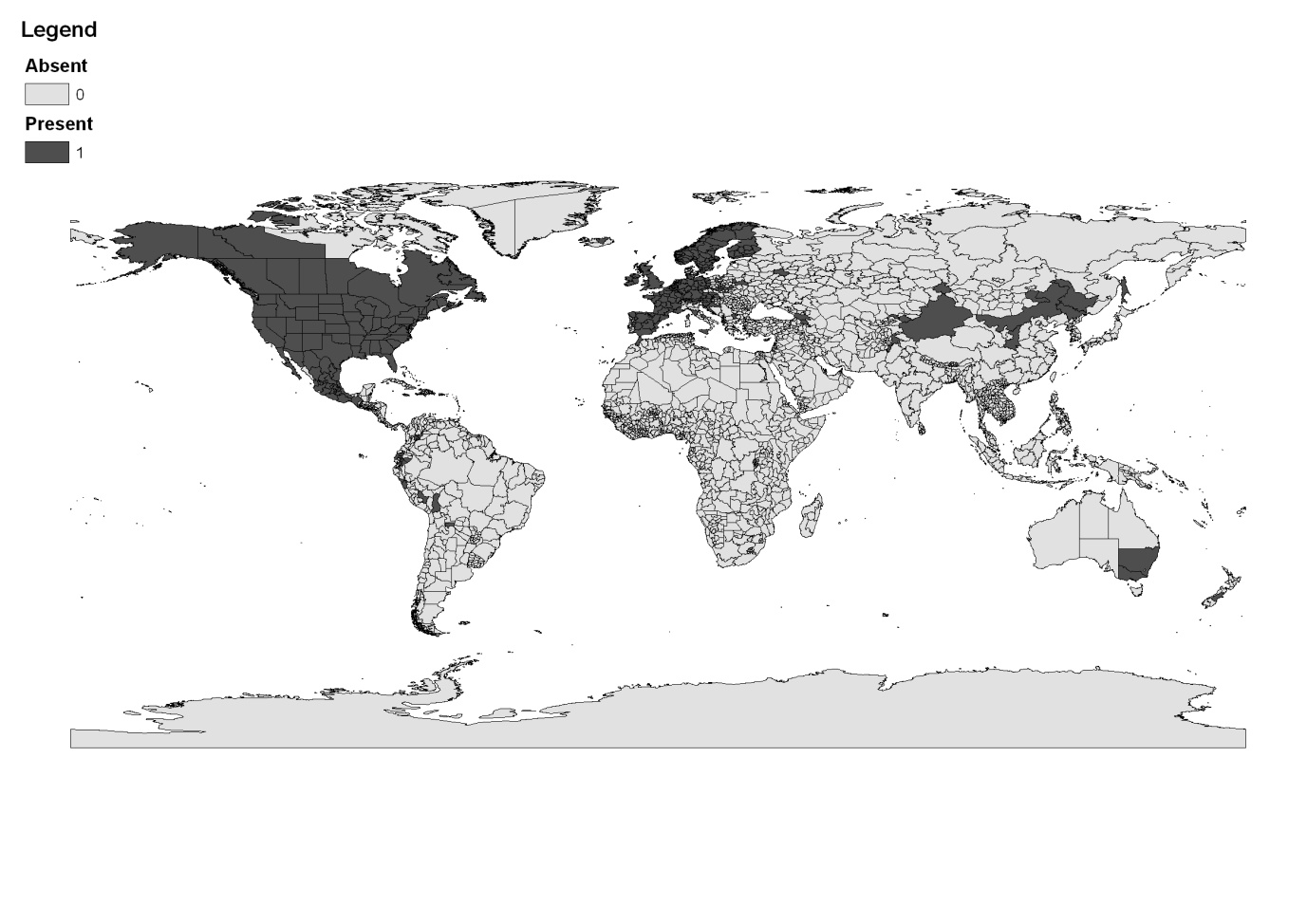

Supplement: Figure S1 — Global recorded distribution of Prunus host plants of R. padi . The distributions of the five recorded Prunus host species were determined using GBIF point records and the USDA PLANTS database (USDA 2010, www.plants.usda.gov) (a). These reports were used to code a world administrative region shapefile (ESRI, Redlands, CA) as present (1), or absent (0) if none of the five Prunus species were not known to occur in the region (b). For New Zealand extra data were gathered that showed that commercial cherries (including P. cerasus) are grown in the Otago, Marlborough and Hawkes Bay regions. (DOCX) [file pone.0040313.s001.docx]
