# Supplementary material for: Modelling the Geographical Range of a Species with Variable Life-History
Source: PLoS One. 2012 Jul 11;7(7):e40313. doi: 10.1371/journal.pone.0040313 (PMC3394791; doi:10.1371/journal.pone.0040313)
Supplement: Supporting Information S1 — More information on the parameterization of CLIMEX models. (DOCX) [file pone.0040313.s002.docx]

## Supporting Information S1: CLIMEX parameters

Further details about the literature supporting the CLIMEX parameters used for the sexual and asexual models (see Table 1).

*Temperature Index*

Initial values for the TI parameters were selected based upon published development rate experimental data. DV0, the minimum temperature for development has been variously reported to range between 4.2 °C [1], 5.8 °C [2] and 8-9 °C [3]. The initial value of 5 °C was adjusted upwards to 6 °C so as to fit better the leading edge of the spring phenological observations from the EXAMINE network station in Finland [4]. The lower (DV1) and upper temperature (DV2) for optimal development were set to 22 and 26 °C respectively, and the upper temperature for development (DV3) set at 30 °C following observations by Dean (1974). Whilst Elliott and Kieckhefer [2] indicate that the upper temperature for development is 25 °C, this appears unlikely given the reports of Asin & Pons [5] on development, and De Barro & Maezler [6] on high temperature survival.

*Moisture Index (MI) (both models)*

The MI parameters were set as follows: SM0 (the minimum soil moisture for development) was set to 0.1 to accord with the permanent wilting point of plants; SM1 and SM2 the lower and upper optimum soil moisture levels were set to 0.5, the soil moisture level when the evapotranspiration rate as a function of pan evaporation starts to decrease significantly, and 1.0 field capacity respectively. SM3 was adjusted upwards to 1.4 in order to allow persistence at known suitable sites in western United Kingdom and northern Irish conditions (Aberystwyth, Ayr, Preston, Rosewarne, Belfast).

*Generation time (both models)*

The PDD parameter in CLIMEX indicates the minimum thermal sum (degree-days above the minimum temperature for development) required to complete a generation. The selected value of 150 °C days was set according to Villanueva and Strong [3] (157 degree-days required for birth to adult). The resulting range boundary defined by the PDD limit agrees with known distribution data.

*Cold Stress (asexual model)* *(CS)*

The CS parameters were fitted using a combination of published cold tolerance information indicating that the nymphs can only tolerate temperatures below -5 °C for a short duration, and known distribution data. Accordingly, TTCS was set to -5 °C and THCS was set to -0.001 Week^-1^ to become lethal after about one month. In order to match the distribution limits of the asexual forms it was also necessary to fit a degree day cold stress mechanism. This form of cold stress accumulates if the species does not experience sufficient heat each week above the developmental threshold. The parameter values of 8 °C days above DV0 (DTCS) and the accumulation rate of -0.001 Week^-1^ (DHCS) were fitted to the known distribution.

*Cold Stress (sexual model)*

A single damaging CS function was fitted to the sexual model. As the species is likely to overwinter as diapausing eggs, reported cold tolerance values were used to select appropriate values for the threshold minimum temperature (-10 °C for TTCS) and the stress accumulation rate (-0.001 Week^-1^ for THCS) [7,8,9]. Whilst nymphs and adults in populations have a good tolerance for cold conditions [see cold stress (asexual model) above], the diapause function is likely to be evolved to protect them from experiencing damaging cold conditions.

*Dry Stress (both models)* *(DS)*

The DS threshold, SMDS, was set to 0.1 using the rationale that if Poaceae hosts were unable to grow due to drought stress, then the population of *R. padi* would similarly suffer stress. The stress accumulation rate HDS was set to become lethal approximately one month after drought stress commenced.

*Wet Stress (both models)* *(WS)*

The WS threshold was set so as to allow persistence at the wettest locations in the United Kingdom and north eastern Ireland. The accumulation rate of 0.01 Week^-1^ (HWS) allowed a good fit in this region.

*Heat Stress (both models) (HS)*

The HS threshold TTHS was set between the upper development threshold, and the instantaneous threshold of 36 °C observed by De Barro & Maelzer [6].

*Diapause (sexual model) (DI)*

The DI was used to simulate the development of sexual reproductive forms and the laying of cold resistant eggs in the woody *Prunus* spp. hosts. DI was set as an obligate winter diapause that caused growth to be switched off but stresses continued to accumulate during this period. The daylength requirement for the onset of diapause was set at 13 hours daylight to approximate comments in Voegtlin & Halbert [10]. The minimum number of days for diapause completion DPD was set to 56 days because an experimental study by Lushai *et al.* [11] noted that the earliest egg hatch occurred 56 days after chilling and post-chilling. The daily minimum temperature to initiate diapause was set at 4 °C as this corresponded well with the daily maximum temperature reported to initiate diapause in some aphids.

**References**

1. De Barro PJ (1991) The ecology of the bird cherry-oat aphid, *Rhopalosiphum padi* (L.) (Hemiptera: Aphididae) in the low rainfall wheat belt of South Australia, PhD thesis, University of Adelaide, Adelaide.

2. Elliott NC, Kieckhefer RW (1989) Effects of constant and fluctuating temperatures on immature development and age specific life tables of *Rhopalosiphum padi* (L) (Homoptera, Aphididae). Canadian Entomologist 121: 131-140.

3. Villanueva BJR, Strong FE (1964) Laboratory studies on the biology of *Rhopalosiphum padi* (Homoptera: Aphidae). Ann Entomol Soc Amer 57: 609-613.

4. Harrington R, Verrier P, Denholm C, Hulle M, Maurice D, et al. (2004) EXAMINE (EXploitation of Aphid Monitoring In Europe): an European thematic network for the study of global change impacts on aphids. In: Simon JC, Dedryver CA, Rispe C, Hulle M editor. Aphids in a New Millennium. pp. 45-49.

5. Asin L, Pons X (2001) Effect of high temperature on the growth and reproduction of corn aphids (Homoptera : Aphididae) and implications for their population dynamics on the northeastern Iberian peninsula. Environmental Entomology 30: 1127-1134.

6. De Barro PJ, Maelzer DA (1993) Influence of high temperatures on the survival of *Rhopalosiphum padi* (L) (Hemiptera, Aphididae) in irrigated perennial grass pastures in South Australia. Australian Journal of Zoology 41: 123-132.

7. Leather SR (1981) Factors affecting egg survival in the bird cherry oat aphid *Rhaopalosiphum padi*. Entomologia Experimentalis Et Applicata 30: 197-199.

8. Lushai G, Hardie J, Harrington R (1996) Inhibition of sexual morph production in the bird cherry aphid, *Rhopalosiphum padi*. Entomologia Experimentalis Et Applicata 81: 117-119.

9. Strathdee AT, Howling GG, Bale JS (1995) Cold-hardiness of overwintering aphid eggs. Journal of Insect Physiology 41: 653-657.

10. Voegtlin DJ, Halbert SE (1998) Variable morph production by some North American clones of *Rhopalosiphum padi* in response to reduced photoperiod and temperature. In: Nieto Nafria JM, Dixon AFG, editors. Aphids in natural and managed ecosystems (Proceedings of the Fifth International Symposium on Aphids, September 15th - 19th, 1997). Leon: Universidad de Leon. pp. 309-315.

11. Lushai G, Hardie J, Harrington R (1996) Diapause termination and egg hatch in the bird cherry aphid, *Rhopalosiphum padi*. Entomologia Experimentalis Et Applicata 81: 113-115.
